# Application of Bevacizumab Combined With Chemotherapy in Patients With Colorectal Cancer and Its Effects on Brain-Gut Peptides, Intestinal Flora, and Oxidative Stress

**DOI:** 10.3389/fsurg.2022.872112

**Published:** 2022-04-11

**Authors:** Chao Chen, Songtao Hou, Fei Zhao, Bin Wu, Tingting Liu, Zhao Zhang, Yuwei Li, Hongchao Li

**Affiliations:** ^1^Department of Colorectal Surgery, Nankai University Affiliated Hospital, Tianjin, China; ^2^Department of Anorectal Surgery, Tianjin Binhai New Area Traditional Chinese Medicine Hospital, Tianjin, China; ^3^Department of General Surgery, Shandong Ningjin People's Hospital, Dezhou, China; ^4^Department of Colorectal Surgery, Hebei Province Hospital of Chinese Medicine, Shijiazhuang, China

**Keywords:** colorectal cancer, bevacizumab, chemotherapy, brain-gut peptides, intestinal flora, oxidative stress

## Abstract

**Objective:**

To investigate the efficacy of bevacizumab combined with chemotherapy in the treatment of colorectal cancer (CRC) and to analyze the effects on brain peptides, intestinal flora, and oxidative stress in CRC patients.

**Methods:**

Eighty two patients with CRC who were admitted to our hospital from March 2018 to June 2021 were selected as the research subjects and divided into the control group (*n* = 41) and the observation group (*n* = 41). The control group was treated with XELOX chemotherapy, and the observation group was additionally treated with bevacizumab, which was repeated every 3 weeks for a total of two treatments. The therapeutic effects of the two groups were evaluated after treatment. The brain-gut peptide index, intestinal flora index and oxidative stress index were detected, and the adverse reactions of the two groups were recorded.

**Results:**

In the control group, ER was 36.59% (15/41) and DCR was 73.17% (30/41). In the observation group, ER was 63.41% (26/41) and DCR was 90.24% (37/41). ER and DCR in the observation group were higher than those in the control group (*P* < 0.05). After treatment, the levels of motilin and gastrin in the observation group were lower than those in the control group, and ghrelin was higher than that in the control group (*P* < 0.05). After treatment, the levels of Bifidobacterium, Lactobacilli and Enterococcus in the observation group were higher than those in the control group, and the level of *Escherichia coli* was lower than that in the control group (*P* < 0.05). After treatment, the SOD level of the observation group was lower than that of the control group, and the MDA level was higher than that of the control group.

**Conclusion:**

Bevacizumab combined with chemotherapy has good efficacy in the treatment of colorectal cancer patients, which can effectively improve the gastrointestinal motility of patients, regulate the intestinal flora of the body, rebuild the microecological balance, effectively reduce the oxidative stress response of patients, and reduce the incidence of adverse reactions.

## Introduction

Colorectal cancer (CRC) is one of the common malignant tumors, ranking the third in the incidence of malignant tumors and the sixth in the mortality rate. The incidence rate of CRC shows a rising trend year by year. Moreover, about one quarter of patients with colorectal cancer are in the advanced stage at the time of their initial diagnosis (1, 2). Surgery is the main treatment, and combined chemotherapy and radiotherapy are the classical treatment modes for colorectal cancer. Although improved chemotherapy regimens and the combination of molecular targeted drugs have increased the survival time of patients, the prognosis of advanced colorectal cancer, especially the right colorectal cancer, is still poor (3, 4). Therefore, exploring effective treatment of CRC is extremely important for improving the survival rate of tumor patients. In recent years, with the deepening of research on the molecular mechanism of tumors, targeted drugs targeting specific tumor molecular markers and signaling pathways have gradually emerged, some of which have been applied in clinical practice, among which the most representative targeted drugs include cetuximab, bevacizumab, and panizumab (5, 6). Vascular endothelial growth factor (VEGF) can specifically bind to the corresponding receptor and act on vascular endothelial cells, and is also an important growth factor to promote angiogenesis. Studies have shown that the inhibition of tumor growth can be achieved by down-regulating the expression of VEGF (7, 8). Bevacizumab can delay tumor growth by specifically binding to VEGF and inhibiting vascular endothelial cell generation and angiogenesis. At present, bevacizumab has been approved for the treatment of advanced CRC, breast cancer and many other advanced malignant tumors (9, 10). The purpose of this study was to investigate the efficacy of bevacizumab combined with chemotherapy in the treatment of CRC and to analyze the effects on brain peptides, intestinal flora and oxidative stress in CRC patients.

## Materials and Methods

### Patients

A total of 82 patients with CRC who were admitted to our hospital from March 2018 to June 2021 were selected as the research subjects. Tumor sites: colon cancer 45 cases, rectal cancer 37 cases. Clinical stages included T3 stage (58 cases) and T4 stage (24 cases). Inclusion criteria: All the patients met the diagnostic criteria of CRC through pathological and cytological examination; Patients who have not received other chemotherapy within 1 month before this treatment; Patients who are not allergic to the current therapeutic drugs; The patient's survival is expected to be more than 3 months; At least 1 target lesion measurable by imaging. Exclusion criteria: Patients with abnormal liver and kidney function; Cardiac insufficiency; Contraindications to chemotherapy; Poor adherence or refusal to participate in the investigator. All the patients were divided into two groups, 41 cases in each group. There was no significant difference in general data between the two groups (*P* > 0.05). As shown in Table 1.

**Table 1:** Comparison of general data between the two groups.

**Group**	**Gender**		**Age (years)**	**Tumor site**		**Clinical stages**		**Tumor diameter (cm)**
	**Male**	**Female**		**Carcinoma of colon**	**Rectal cancer**	**T3**	**T4**	
Control group (*n* = 41)	26	15	49.38 ± 8.76	25	16	31	10	3.49 ± 0.58
Observation group (*n* = 41)	22	19	49.66 ± 8.43	20	21	27	14	3.54 ± 0.61
t/χ^2^	0.804		0.147	1.231		0.943		0.380
P	0.369		0.883	0.267		0.332		0.705

### Treatment Methods

The control group was treated with XELOX chemotherapy: On the first day, oxaliplatin (Qilu Pharmaceutical Hainan Co., LTD.) 130 mg/m^2^ was given by intravenous infusion. Capecitabine (Shanghai Roche Pharmaceuticals Co., LTD.) 1,000 mg/m^2^ was administered orally twice daily for 14 consecutive days. Three weeks is one cycle. On the basis of the control group, bevacizumab was additionally used in the observation group: bevacizumab (Roche Diagnostics GmbH) was given by intravenous infusion at 7.5 mg/kg. The treatment was repeated every 3 weeks and the curative effect was observed after 2 cycles.

### Observation Indicators

#### Efficacy Evaluation

The efficacy of patients after treatment is evaluated according to the efficacy evaluation criteria of solid tumors (11), which can be divided into complete response (CR), partial response (PR), disease stability (SD), and disease progression (PD). The effective rate (ER) = (CR + PR) cases/total cases ×100%, and the disease control rate (DCR) = (CR + PR + SD) cases/total cases ×100%.

#### Detection of Brain-Gut Peptide Indicators

Five milliliter of fasting venous blood in the morning of the patients was drawn before and after treatment, and the serum levels of motilin, gastrin and ghrelin of the patients were detected by radioimmunoassay. The relevant kits were purchased from Shanghai Yuanxin Biotechnology Co., Ltd.

#### Detection of Intestinal Flora Indicators

Anaerobes: *Bifidobacterium* and *Lactobacilli*; Aerobic bacteria: *Escherichia coli* and *Enterococcus*. Patients' fresh feces (0.1 g) were collected before and after treatment and diluted with normal saline at a ratio of times and mixed evenly, and inoculated into different media. The anaerobes were cultured by air extraction and ventilation for 72 h and then counted. The aerobic bacteria were cultured at 37°C for 48 h and then counted. They were identified by ALB semi-automatic microbial assay system.

#### Detection of Oxidative Stress Indicators

Serum superoxide dismutase (SOD) level was determined by xanthine oxidase colorimetric method, and serum malondialdehyde level was determined by thiobarbituric acid colorimetric method. The relevant kits were purchased from Shenzhen Jingmei Biological Technology Co., Ltd.

#### Adverse Reactions

The adverse reactions such as thrombocytopenia, nausea and vomiting, hypertension, and rash in the two groups were recorded during the treatment.

### Statistical Methods

All data were processed with SPSS 22.0 statistical software. The enumeration data were examined by *X*^2^-test and expressed by [*n* (%)], the measurement data were examined by *t*-test and expressed by (x ±s). The difference is statistically significant when *P* < 0.05.

## Results

### Comparison of Efficacy Between the Two Groups

**Figure 1:**
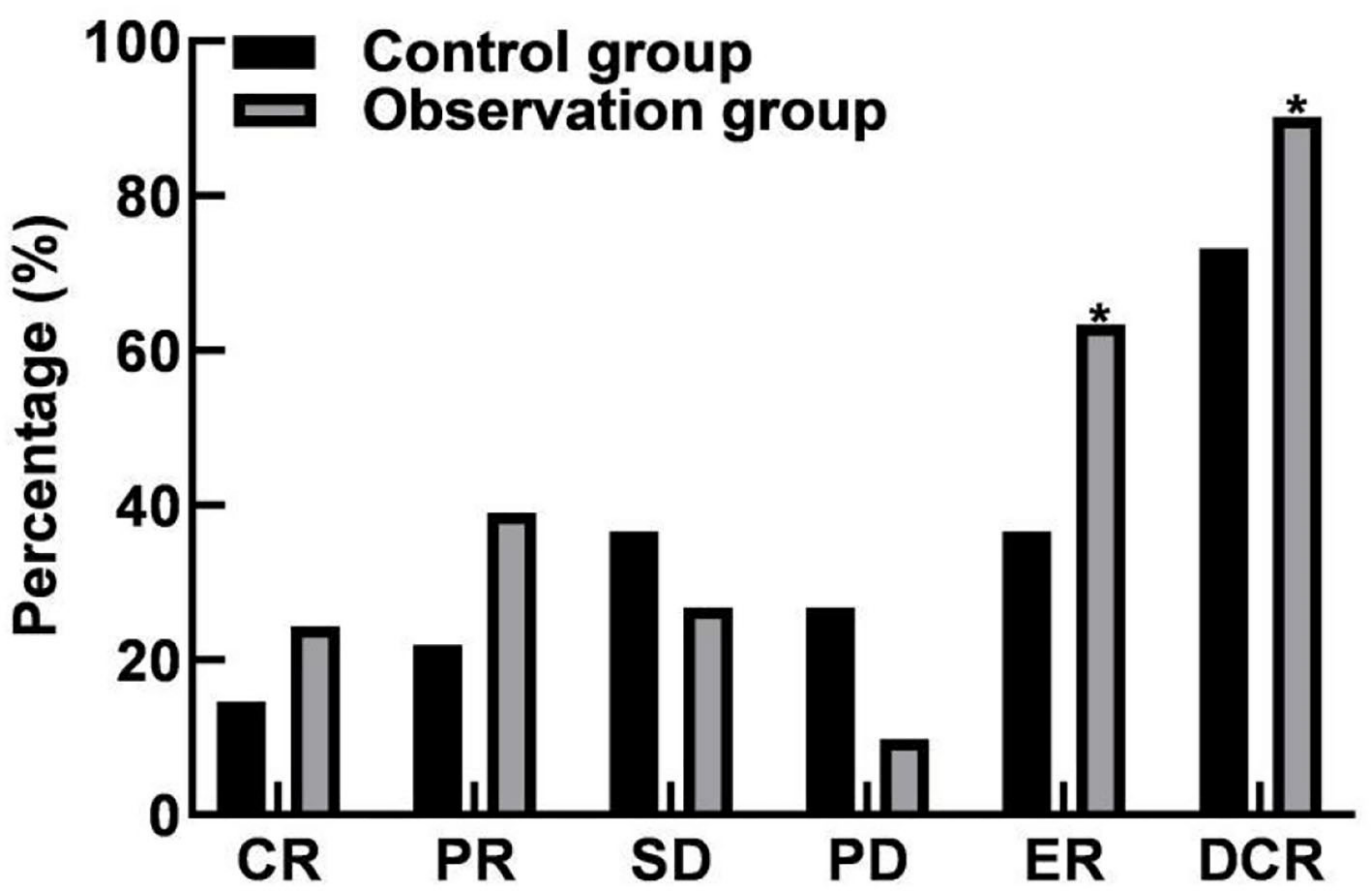
Comparison of efficacy between the two groups. Compared with the control group, *P < 0.05.

In the control group, ER was 36.59% (15/41) and DCR was 73.17% (30/41). In the observation group, ER was 63.41% (26/41) and DCR was 90.24% (37/41). ER and DCR were significantly different between the two groups (*P* < 0.05). As shown in Figure 1.

### Comparison of Brain-Gut Peptide Indicators Between the Two Groups

**Figure 2:**
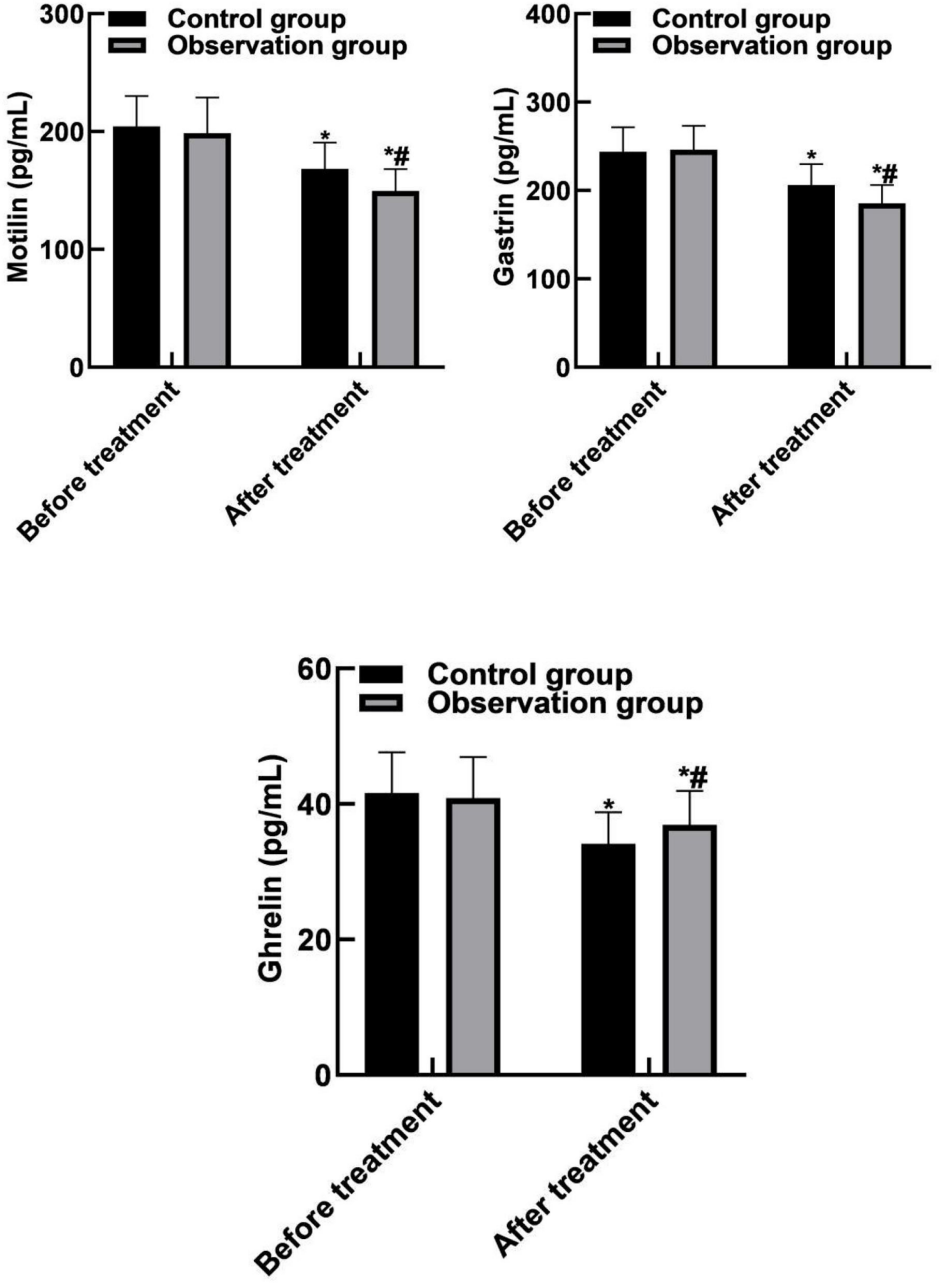
Comparison of brain-gut peptide indicators between the two groups. Compared with before treatment, *P < 0.05. Compared with the control group, ^#^*P* < 0.05.

The levels of motilin, gastrin and ghrelin in control group were (168.45 ± 22.16) pg/mL, (206.18 ± 23.63) pg/mL, and (34.12 ± 4.63) pg/mL, respectively. Observation group were (149.58 ± 18.63) pg/mL, (185.65 ± 20.49) pg/mL, (36.94 ± 5.01) pg/mL, respectively. There were significant differences in motilin, gastrin and ghrelin levels between the two groups after treatment (*P* < 0.05). As shown in Figure 2.

### Comparison of Intestinal Flora Indicators Between the Two Groups

**Figure 3:**
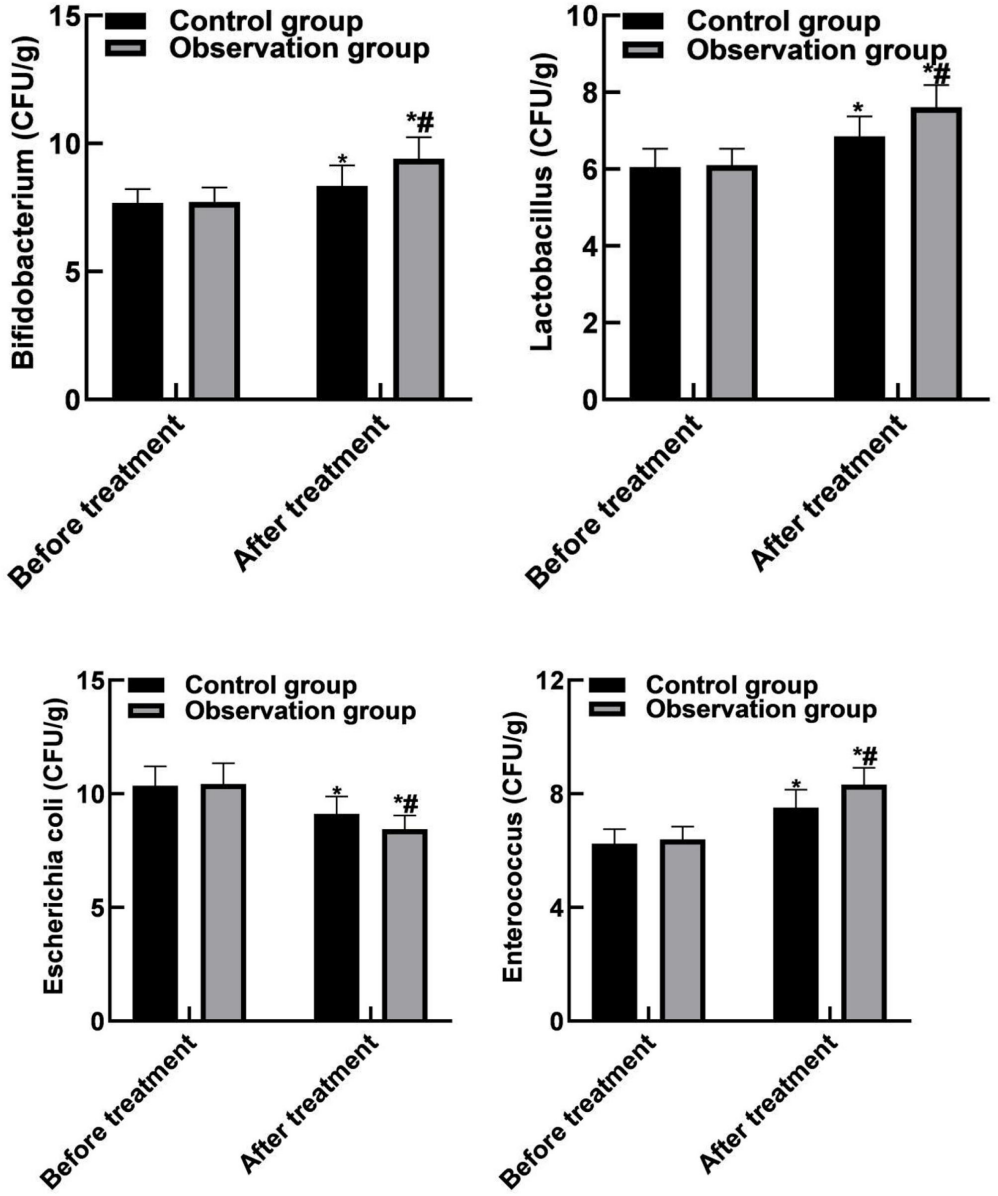
Comparison of intestinal flora indicators between the two groups. Compared with before treatment, *P < 0.05. Compared with the control group, ^#^*P* < 0.05.

After treatment, the levels of bifidobacterium, Lactobacillus and enterococcus in control group were (8.35 ± 0.79) CFU/g, (6.85 ± 0.52) CFU/g, (9.12 ± 0.76) CFU/g, and (7.51 ± 0.63) CFU/g, respectively. Observation group were (9.41 ± 0.83) CFU/g, (7.61 ± 0.58) CFU/g, (8.45 ± 0.59) CFU/g, (8.32 ± 0.59) CFU/g, respectively. There were significant differences in the levels of bifidobacteria, lactobacillus, *Escherichia coli*, and enterococcus between the two groups after treatment (P < 0.05). As shown in Figure 3.

### Comparison of Oxidative Stress Indicators Between the Two Groups

**Figure 4:**
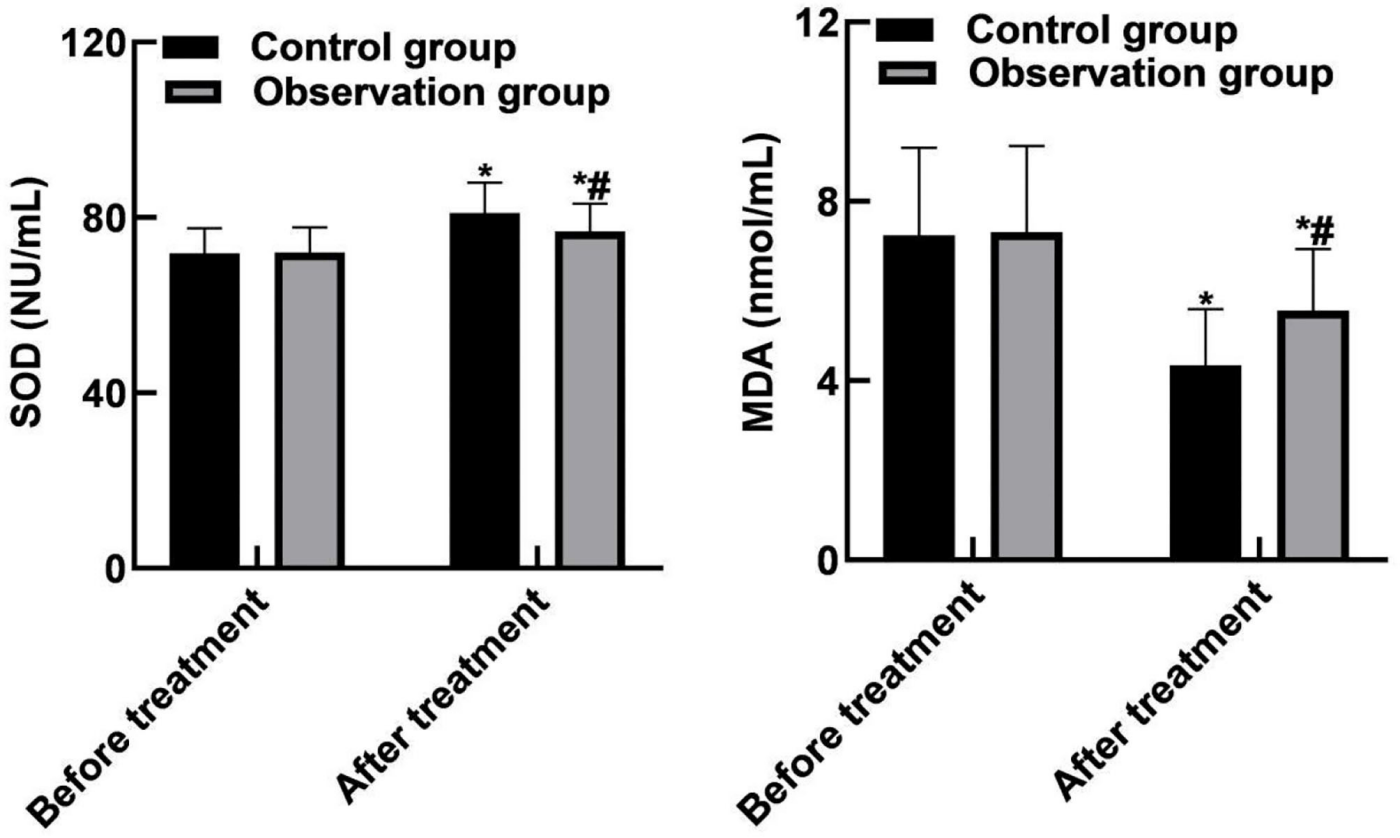
Comparison of oxidative stress indicators between the two groups. Compared with before treatment, *P < 0.05. Compared with the control group, ^#^*P* < 0.05.

SOD and MDA levels in the control group were (81.05 ± 6.91) NU/mL and (4.35 ± 1.24) nmol/mL, respectively. The observation group were (76.83 ± 6.32) NU/mL and (5.56 ± 1.38) nmol/mL, respectively. SOD and MDA levels were significantly different between the two groups after treatment (*P* < 0.05). As shown in Figure 4.

### Comparison of Adverse Reactions Between the Two Groups

**Figure 5:**
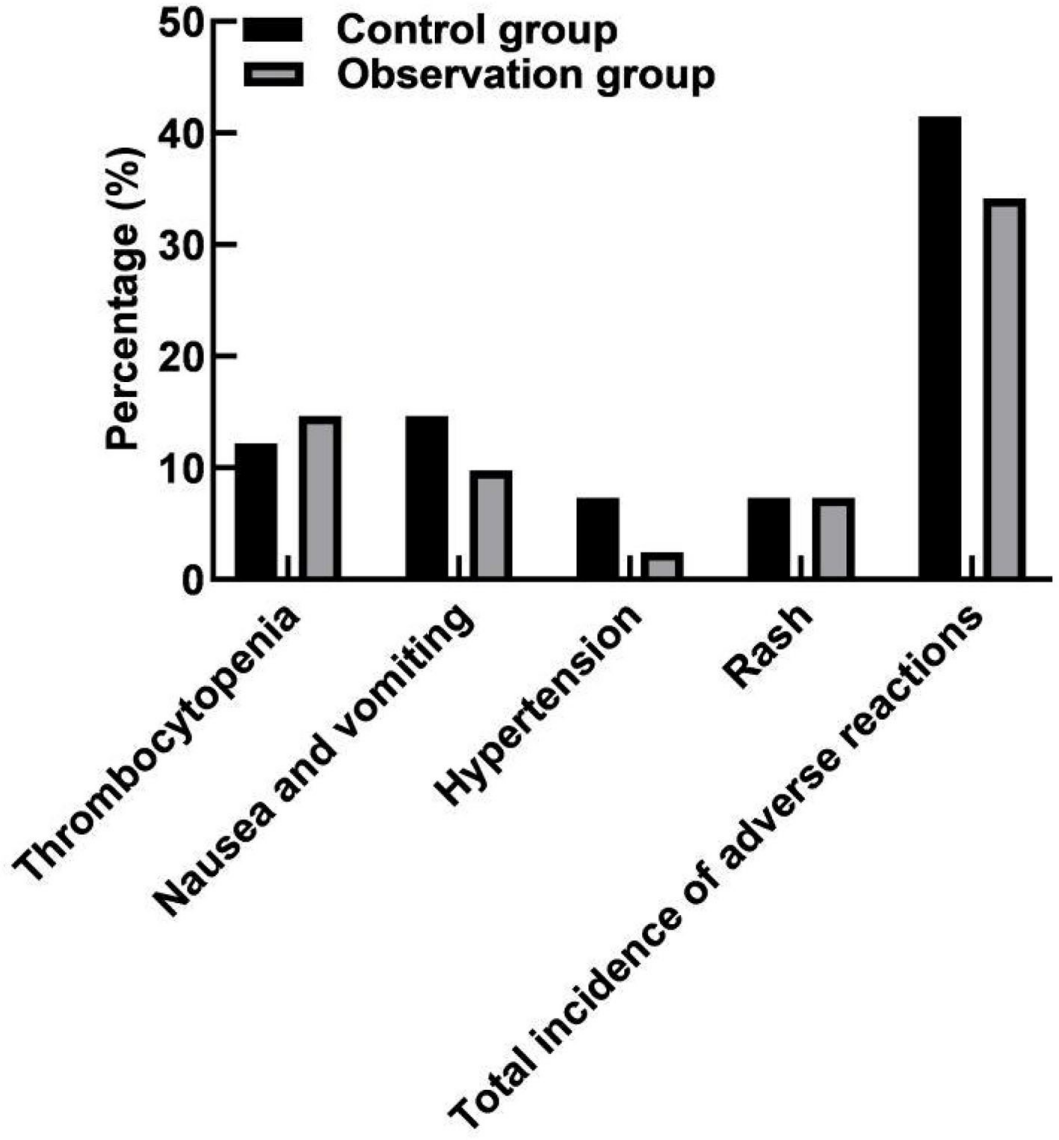
Comparison of adverse reactions between the two groups.

The incidence of adverse reactions was 41.46% (17/41) in the control group and 34.15% (14/41) in the observation group. There was no significant difference in the total incidence of adverse reactions between the two groups (*P* > 0.05). As shown in Figure 5.

## Discussion

Colorectal cancer is a common malignant tumor in the gastrointestinal tract. It has a high incidence and mortality in the elderly, which are mostly related to the high-fat diet, large intestinal adenoma and related genetic factors (12, 13). The main clinical symptoms of CRC are hematochezia, increased stool frequency, internal urgency and then severity, which have seriously affected the life and health of patients as well as the quality of daily life (14, 15). Patients with early colorectal cancer have no specific symptoms, so they can be easily ignored to delay the optimal treatment timing. With the progression of the disease, symptoms such as abdominal pain, anemia, abdominal mass and hematochezia will gradually appear. Most patients have progressed to the intermediate and advanced stage when they are diagnosed, and the cancer cells metastasize to a distance, so their lives can only be prolonged by chemotherapy (16, 17). At present, the oxaliplatin-based combination regimen of XELOX and FOLFOX is mainly used for patients with advanced colorectal cancer who miss the opportunity of surgery. Compared with the latter, the XELOX regimen has less toxic and side effects and is widely used in clinical practice. Relevant clinical findings show that oxaliplatin chemotherapy has a significant clinical effect, which can remove more than 25% of cancer cells and surrounding lesions in patients (18, 19). However, oxaliplatin chemotherapy also has some shortcomings. Due to many adverse reactions after chemotherapy, especially liver function damage and fatigue, the follow-up recovery time of patients is prolonged, and thus the normal life of patients is affected (20, 21). Therefore, this result will increase the suffering of the patient and the recurrence rate after treatment, seriously affecting the patient's mood and aggravating the deterioration of the disease. Capecitabine, as a new fluorouracil drug, has the characteristics of selective and specific targeting. After drug administration, capecitabine is converted into an intermediate 5-deoxy−5-fluorocytidine in the liver, and catalyzes the formation of fluorouracil in lesion tissues to directly act on cancer cells. The capecitabine has the characteristics of rapid absorption, strong pertinence and high safety. Bevacizumab, as a monoclonal antibody drug, can directly act on vascular endothelial growth factor to inhibit endothelial cell proliferation and angiogenesis, so as to achieve the purpose of inhibiting the growth of lesions.

Angiogenesis is one of the ten major features of tumors, and angiogenesis in tumors can provide the nutrients needed for tumor growth, so as to maintain the continuous proliferation of tumors. In the angiogenesis process, there are many angiogenic factors involved, among which VRGF belongs to endothelial cell-specific factor and is one of the most potent angiogenic factors (22, 23). VEGF mainly binds to VEGFR2 and activates the downstream signaling pathway, ultimately leading to the formation of new blood vessels. The main functions of VEGF: (1) Specifically enhance the mitosis of vascular endothelial cells, stimulate the proliferation of vascular endothelial cells and promote neovascularization; (2) Improve the permeability of micro blood vessels, and provide nutrients for the growth of tumor cells and the establishment of capillary network through the extravasation of nutrients such as plasma macromolecules. There is often a relatively high expression of VEGF in colorectal cancer patients, and relevant studies have also shown that high expression of VEGF in tumor tissue or blood often indicates a poor prognosis (24, 25). At present, the treatment targeting VEGF/VEGFR has become an important means of tumor treatment. The mechanism is mainly through competitive binding with endogenous VEGF, and inhibiting or reducing the binding of VEGF to vascular endothelial cell surface receptors, thereby inhibiting endothelial cell proliferation and angiogenesis, and finally playing a role in inhibiting tumor growth (26, 27).

The results of this study showed that the ER and DCR values in the observation group were higher than those in the control group, indicating that bevacizumab combined with chemotherapy could effectively improve the treatment effect. The reason is analyzed that bevacizumab can affect the proliferation of endothelial cells and inhibit the formation of tumor neovascularization. It is an antibody drug approved in the world for inhibiting the growth of blood vessels. Bevacizumab takes vascular endothelial growth factor as a target, reduces neovascularization, promotes the degradation of the existing tumor blood vessels, blocks oxygen, blood and other nutrients for tumor growth, inhibits endothelial cell mitosis, leads the surviving tumor blood vessels to tend to be normal, limits the growth of tumors, and has obvious effects on the treatment of various metastatic cancers. Gastrointestinal movement is jointly regulated by a variety of mechanisms such as vegetative nervous system, myogenic electrical activity and body fluid. Brain-gut peptides are biologically active enzymes with hormone-like effects, and they participate in the motility regulation of digestive organs together with the nervous system. Motilin is mainly secreted by M cells of duodenum and jejunum, and its main effect is to induce transitional motor complex waves during digestion and accelerate the emptying of the gastrointestinal tract. Gastrin is secreted by G cells in gastric antrum and duodenum, which can stimulate the secretion of gastric acid and pepsin, and promote the gastrointestinal motility and the growth of gastric mucosa (28, 29). Ghrelin is mainly secreted by the stomach and has the effects of promoting growth hormone, stimulating appetite, increasing body weight, and regulating energy metabolism. The results of this study showed that after treatment, the levels of motilin and gastrin in the observation group were lower than those in the control group, and ghrelin was higher than that in the control group. It is suggested that bevacizumab combined with chemotherapy can improve gastrointestinal motility.

In human intestinal bacteria, obligate anaerobic bacteria such as bifidobacterium, lactobacillus, and bacteroides account for about 99% of that total intestinal bacteria, and facultative anaerobe such as Escherichia coli and Enterococcus account for about 1%, constituting a complex intestinal micro-ecological system. Bifidobacterium and Lactobacillus belong to beneficial bacteria, while Escherichia coli and Enterococcus belong to harmful bacteria. The coordination effect of intestinal beneficial bacteria can promote intestinal peristalsis and mucus flow, resist the adhesion of harmful bacteria to epithelial cells, and then form the intestinal mucosal barrier function, and regulate the intestinal mucosal immune system (30, 31). The results of this study showed that after treatment, the levels of Bifidobacterium, Lactobacilli and Enterococcus in the observation group were higher than those in the control group, and the level of Escherichia coli was lower than that in the control group. These results indicated that bevacizumab combined with chemotherapy could significantly improve the intestinal flora of patients and rebuild the intestinal microecological balance. Besides, the results of this study showed that after treatment, the SOD level of the observation group was lower than that of the control group, and the MDA level was higher than that of the control group. These results indicated that bevacizumab combined with chemotherapy can reduce the stress response of patients.

The toxic and side effects of chemotherapy drugs will lead to bone marrow suppression, gastrointestinal and skin abnormal reactions in patients, while bevacizumab can also lead to internal bleeding in target organs, gastrointestinal perforation and other adverse reactions. There was no statistical difference in the incidence of adverse reactions between the two groups in this study. In clinical treatment, attention should be paid to adverse reactions in patients, and preventive measures should be formulated in advance to improve the quality of life of patients.

## Conclusion

Bevacizumab combined with chemotherapy has good efficacy in the treatment of CRC patients, which can improve the gastrointestinal motility of patients, regulate the intestinal flora of the body, rebuild the microecological balance, effectively reduce the oxidative stress response of patients, and reduce the incidence of adverse reactions.

## Data Availability Statement

The original contributions presented in the study are included in the article/supplementary material, further inquiries can be directed to the corresponding authors.

## Ethics Statement

The studies involving human participants were reviewed and approved by the Ethics Committee of Nankai University Affiliated Hospital. The patients/participants provided their written informed consent to participate in this study.

## Author Contributions

All authors of the study made equal contributions, including the design of the study, conduct of the experiments, evaluation of the results, statistics of the data, and writing of the article. All authors contributed to the article and approved the submitted version.

## Funding

This study was supported by Science and Technology Project of Tianjin Binhai New Area Health and Family Planning Commission (Grant No. 2018BWKQ031), Foundation of Tianjin Municipal Health Commission (Grant No. ZC20097), and Foundation of Tianjin Union Medical Center (Grant Nos. 2020YJ017 and 2017YJZD005).

## Conflict of Interest

The authors declare that the research was conducted in the absence of any commercial or financial relationships that could be construed as a potential conflict of interest.

## Publisher's Note

All claims expressed in this article are solely those of the authors and do not necessarily represent those of their affiliated organizations, or those of the publisher, the editors and the reviewers. Any product that may be evaluated in this article, or claim that may be made by its manufacturer, is not guaranteed or endorsed by the publisher.
